# Nanostructured Biomaterials and Their Applications

**DOI:** 10.3390/nano3020242

**Published:** 2013-05-10

**Authors:** Kirsten Parratt, Nan Yao

**Affiliations:** Princeton Institute for the Science and Technology of Materials, Princeton Imaging and Analysis Center, Princeton University, 120 Bowen Hall, Princeton, NJ 08544, USA; E-Mail: kparratt@princeton.edu

**Keywords:** nacre, nanoparticles, hydroxyapatite, drug delivery, nanostructured scaffolds, biomimetics

## Abstract

Some of the most important advances in the life sciences have come from transitioning to thinking of materials and their properties on the nanoscale rather than the macro or even microscale. Improvements in imaging technology have allowed us to see nanofeatures that directly impact chemical and mechanical properties of natural and man-made materials. Now that these can be imaged and quantified, substantial advances have been made in the fields of biomimetics, tissue engineering, and drug delivery. For the first time, scientists can determine the importance of nanograins and nanoasperities in nacre, direct the nucleation of apatite and the growth of cells on nanostructured scaffolds, and pass drugs tethered to nanoparticles through the blood-brain barrier. This review examines some of the most interesting materials whose nanostructure and hierarchical organization have been shown to correlate directly with favorable properties and their resulting applications.

## 1. Introduction

As technology evolves to let us examine materials on smaller and smaller length scales, it follows that we would also develop techniques to manipulate the structure and composition on the same. These innovations are especially visible in the field of material engineering, where it is desirable to understand every aspect of a structure as well as in the life sciences where we want to match the complexity demonstrated in the human body. The body is capable of arranging proteins and other molecules with nano-precision, so engineers looking to produce materials that interact with the body would like to do the same. While many research questions have benefited from the advent of well-understood nanomaterials, this work will focus on topics that have used nanoengineering to directly improve the function of materials in the body. These include the structure and failure modes of nacre, one of nature’s most impressive composites with manifold applications to the field of biomimetics; bioactive glass nanoparticles, one of the most promising components in modern tissue engineering; hydroxyapatite fibers, which can integrate immediately with the body; and biodegradable nanospheres, which can transport drugs across the notoriously strict blood-brain barrier. Transitioning to the nanoscale results in increased surface complexity which then causes dramatic improvement in characteristics such as cell adhesion, surface friction, fracture behavior, and fibroblast growth. Nanomaterials have also been transformative in drug delivery systems as they can be tuned to inject smaller but more effective payloads directly to the damaged site. For all of the above systems, this work will explain how the life sciences have benefited from the introduction of nanomaterials and what important structural properties impact their function. We will then discuss how nanoengineering has transformed these materials’ life science applications.

## 2. Biomaterials

### 2.1. Nacre

One of the most studied natural materials in the field of biomimetics is nacre which is the mother-of-pearl found inside many seashells*.* Nacre is an especially interesting material for study because it is composed of 95% calcium carbonate, in the form of aragonite, layered with 5% of polymeric organic matter, and yet it has a fracture strength of about 3000 times pure calcium carbonate [1]. The nature of this improvement in mechanical strength is related to nacre’s unique microstructure and yields many suggestions for how to improve man-made materials. While the strength of a composite can be improved by striving for stronger individual components, nacre stands as an example of how careful placement of weaker materials can yield similar results. It has also been pointed out that nacre has many characteristics that are desirable in biomedical materials. The many components of nacre have a hierarchical organization, mild processing conditions, simple constituents, durable interfaces, viscoelastic properties, good fatigue performance, and some extent of self-healing [2]. Incorporating these qualities in biomaterials is therefore a desirable goal and careful study of nacre’s structure and formation can help achieve it.

**Figure 1:**
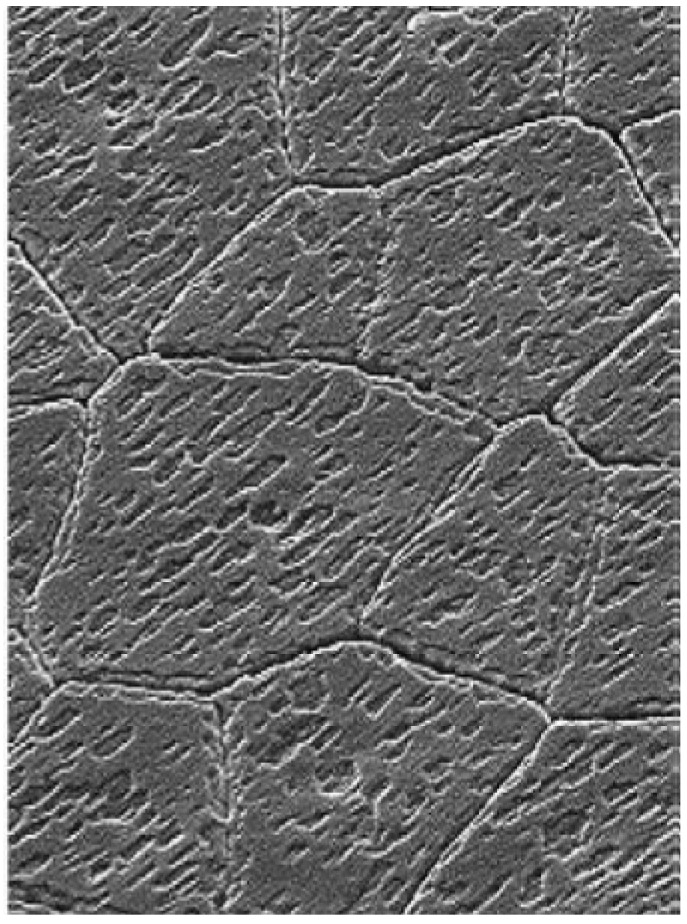
SEM image showing that the imprints of nano-asperity grooves correspond to the crystal directions of the aragonite platelets. Reproduced from [2] with permission from The Royal Society.

In nacre, the calcium carbonate is present as aragonite tablets of about 5 µm across and 0.5 µm thick [2]. These tablets can then be further sectioned as numerous nanograins of approximately 10–50 nm in diameter held together by an organic matrix [2]. Individual aragonite platelets grow between polymer sheets in the organic matrix via the assembly of nanoparticles nucleated from colloidal amorphous calcium carbonate [3,4]. These nanograins are capable of many of the same deformation behaviors observed to occur between the constituent tablets on the microscale such as deformation and rotation [3]. Natural nacre is structured in two different forms: columnar and sheet, which are distinguished based on the orientation of the centers of successive platelets stacked on top of one another, and are located in the shell for optimal performance [5]. In both forms, there are layers about 300 µm thick composed of sublayers of aragonite platelets which are separated by organic layers of 20–50 nm [6]. These 300 µm platelet layers are separated by thicker 20 µm mesolayers of multiple organic layers [2]. The thick mesolayers are a result of seasonal effects as changes in the feeding patterns limit available ions for mineral formation [4]. Within each aragonite layer, there are large domains of platelets which have the same crystallographic orientation (Figure 1) [1].

This alignment between platelets is also preserved in the vertical direction as proved by identical pole figures at 5 and 10 degrees, which corresponds to a depth of 5–11 µm or up to 20 platelet layers [2]. Yao *et al*. has proposed that these aragonite layers grow via a spiral helix propagation model which explains the large-scale alignment of the platelets in both the lateral and vertical directions [2]. The sources of these helices are growth fronts which are anchored by paired screw dislocations on each end to form an extended line defect with a thickness of one platelet layer [2].

The characteristics of these aragonite platelets are only part of the explanation of why nacre is so impressive. It is also important to consider the mechanical and chemical properties of the organic material in the shells. The organic mesolayers are made of about 7% oriented beta-chitin fibrils, which are silk-like proteins organized into sheets [7]. These have been found to extend over multiple platelets and is constructed of individual chitin fibers of lengths of at least1µm linked into a network by proteins [7]. These fibrils are then surrounded by about 75% proteins, including lustrinA which is composed of many sacrificial loops that can be extended during stress periods [5,7]. Since these loops are created through the van der Waals association of hydrophilic or hydrophobic domains, new loops can be formed after deformation to restore some of the mechanical properties [8]. The interfaces between the tablets and organic layer are anchored assemblies of peptide chains, mostly amino acids with carboxylic groups [9]. Finally, the organic material is found even within the platelets themselves in the form of discontinuous layers of aspartic acid-rich glycoproteins called the “intratabular matrix” [7,9].

The organic matrix performs many roles in the shell and recently it has been posited that the organic layer does more to organize the material structure than the inorganic. Organic layers are laid down by the abalone with a regular frequency such that tablets sandwiched between them can grow only until their height is arrested. However, growth can still occur laterally within this layer as material passes through organic pores until all the space has been filled [10]. This material probably passes to the tablet in the form of ions or amorphous calcium carbonate [11]. As noted before, large domains of similarly oriented crystals are common in both the vertical and horizontal direction. A long-standing question has therefore been how this orientation is transferred from one platelet to another in the absence of crystal continuity. Wise and deVilliers hypothesized that the organic layer was in fact oriented by the platelets over which it was deposited [12]. This in turn could explain how crystal orientation is then transferred again to the platelets that grow overtop of it. However, other authors have recently suggested that the organic layer is disordered and tablets may pass crystallographic material from one layer to the next in order to maintain this orientation either through crystalline continuities or as amorphous calcium carbonate. However, before we can compare these theories, we need an understanding of how the organic layer functions.

**Figure 2:**
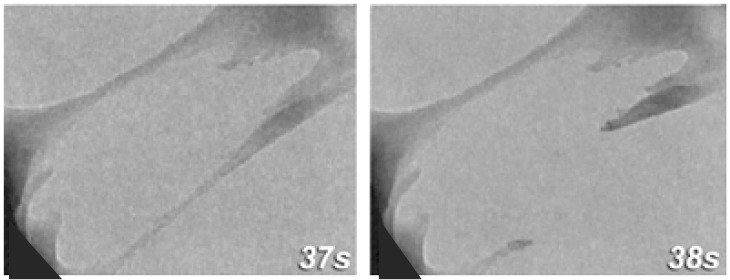
TEM still image sequence showing *in situ* deformation of organic matrix between plates with the time intervals shown in seconds. Adhesion at the wall is strong and failure will occur by deformation of the ligament. The recoiling broken strand shows densification at its base. Reproduced from [8] with permission from Cambridge University Press.

While this explains what nacre is composed of, it does not explain its impressive mechanical strength. An individual aragonite tablet has a mechanical strength approximately the same as that of pure calcium carbonate [8], so what about nacre gives the notable improvement? First, consider the effect of aragonite nanograins and platelets on the shell’s deformation. Cross-sections of nacre show that the platelets and organic matrix are arranged in a “brick-and-mortar” pattern. This severely limits the ability of any crack to propagate perpendicular to these layers; it will quickly run directly into one of the tablets. Not only are these tablets strong due to the innate material characteristics, but the dimensions of the platelets are such that they ensure optimum strength and flaw tolerance [9,11,13]. The edges of the tablets are slightly thicker than the center so that as the tablets try to slide past one another, they find it increasingly difficult to move due to the difficulties of shifting all the surrounding platelets out of formation and will become locked together [8]. However, under tension, failure of the aragonite layer will occur through tablet pull-out and relies on the organic layer for durability [5,11]. At the platelet level, Sumitomo *et al*., found that the organic matrix could stretch into ligaments that would reach 460 nm before rupturing and the two broken ligament ends would then densify with reformation (Figure 2) [8]. The organic material is less likely to pull away from the tablet because the lustrinA present in the system adheres the chitin cores to the mineral tablets [11]. By measuring the force-extension behavior of the organic material, they found that it matched the behavior expected for the reversible unfolding of modular domains and sacrificial bonds [2].

Organic ligaments can be seen bridging the gap between separated platelets as well as between the nanograins of fractured platelets [3]. In the case of the nanograins, there is a high degree of overlap and the organic matrix is at most 10 nm thick [3]. This organic material can stretch to 40 nm before failure and probably occurs in the platelet because it was trapped between nucleating nanograins during crystallization [3].

**Figure 3:**
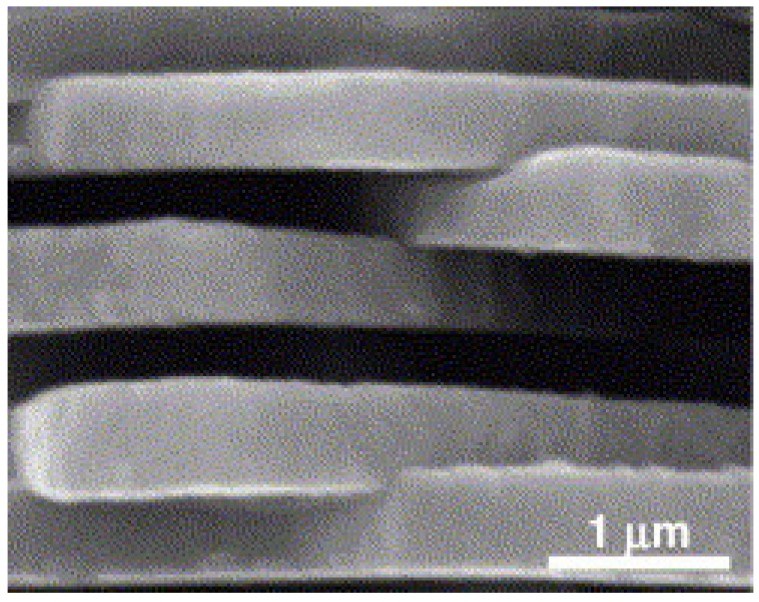
SEM image of a fractured nacre surface showing presence of interlocking between platelets of nacre responsible for its mechanical response. Reproduced from [14] with permission from Elsevier.

It has been proposed that individual platelets could also interact in other ways to prevent deformation including shear resistance from the asperities, mineral bridges that dissipate energy by cracking, and crack-tip shielding due to the integration of two materials with different elastic moduli [9]. The shear resistance from asperities and interlocks has been modeled by using finite elements by Katti (Figure 3), who proved that these were the dominating sources of friction for the interface and also acted to prevent catastrophic failure [2].

Interlocks were estimated to reach a depth of 50 nm (20 nm of which constituted the organic separating layer) and created by adjacent platelets being rotated approximately 5 degrees with respect to one another [14]. Katti’s simulation found that nacre without interlocks had a yield stress of 5 MPa but nacre with interlocks had a yield stress of 37 MPa, and therefore concluded that interlocks are an essential part of nacre’s toughening mechanism [14]. By performing indentation tests, Katti’s group also found that the hardness and elastic modulus values decrease with increasing load [15]. They attribute this to crack propagation as the different loads will engage the organic and inorganic material to different degrees [15].

**Figure 4:**
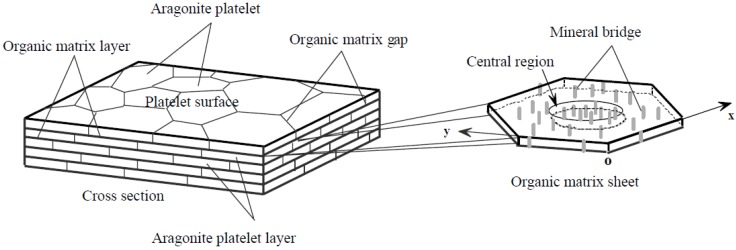
Schematic illustration showing the “brick and mortar” microarchitecture of nacre; the mineral bridges in an organic matrix sheet. While we are considering the mineral bridges to in fact be nanoasperities, the image shows the pores in the organic layer and therefore the arrangement of these nanoasperities. Reproduced from [16] with permission from Elsevier.

Mineral bridges are often cited as a logical source of resistance to shear forces because they would require an energy input sufficient to crack them before platelets can slide past one another. These are discussed in detail by Song *et al.*, who describe the existence of nanopores in the organic matrix and attribute them to the presence of mineral bridges (Figure 4). While these pores are more likely due to jutting nanoasperities on the platelet surfaces, they still are one possible explanation for how crystallographic information is transferred from one layer to the next [2].

**Figure 5:**
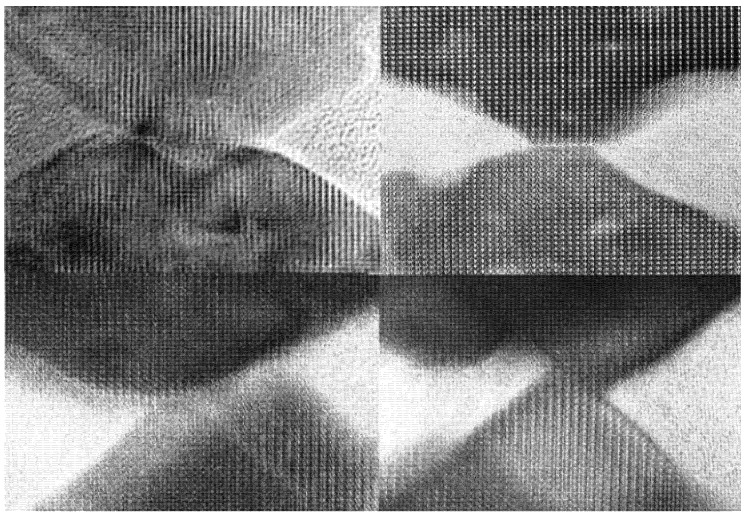
Demonstrates that crystal outgrowths of nano-asperities from the top and bottom platelets are not exactly connected or epitaxial, even though they share the same crystal orientation as indicated by atomic lattice alignments. Reproduced from [2] with permission from The Royal Society.

These nano-pores were also found to cluster in the center of platelets and be sparser on the edges; this greatly improves nacre’s ability to deflect cracks. Instead of cracks being allowed to propagate in the organic matrix layer, which would delaminate the platelets, it will propagate in the matrix layer only until it reaches the nanoasperities before being deflected and traveling down the next organic matrix gap [16]. This allows the organic layer to adsorb much of the incoming energy and will minimize the degree of deformation. While Song *et al*.’s findings regarding nanopores and subsequent predictions about crack propagation still hold true, new research has shown that these “mineral bridges” are not in fact continuous. Yao *et al*. used transmission electron microscopy to prove that the bridges that had been previously referenced were in fact abutting asperities and not continuous crystal bridges (Figure 5) [2]. This was confirmed by Checa *et al*., who found that what appear to be mineral bridges are simply overlapping crystal extrusions through large pores in the organic matrix [17].

**Figure 6:**
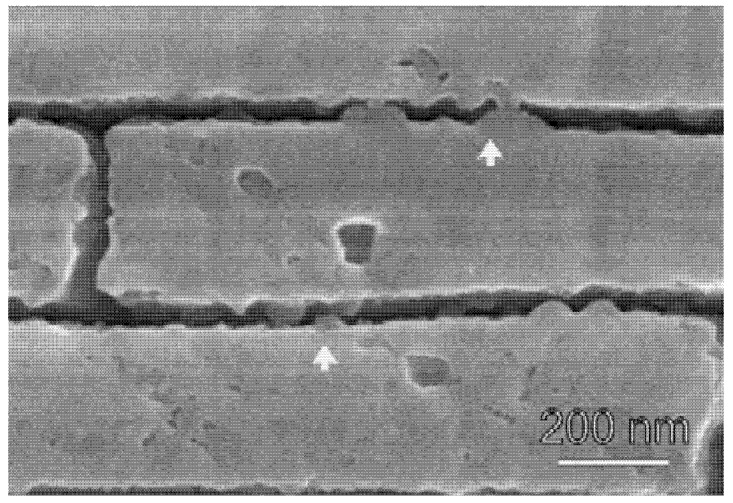
Cross-section of abalone nacre showing the detailed structure at the lamellae boundaries with arrows to highlight some of the locations where nanoasperities interpose. As the platelets shift past one another, the energy to move these nanoasperities over each other will contribute to the dissipation of energy. Reproduced from [5] with permission from Cambridge University Press.

Therefore, while energy would be required to shift the nanoasperities past one another, the movement will require less than if breaking a mineral bridge was required. To sum these contributions, Lin and Meyers measured the shear strength of the interface between platelets to be about 50 MPa with an average maximum shear strain of 0.3 [6]. Wang *et al.* have done the same for compression and tensile testing. For compression, they found a stress of 370 MPa and a strain of 0.005 [5]. For tensile testing, the stress was measured to be 105 MPa and the strain as 0.011 [5]. These tests can be interpreted give an idea of nacre’s behavior in under each condition and emphasizes the importance of the organic-inorganic interactions. These behaviors have also been replicated in simulations by Tang *et al*. [18]. In the case of shear, nacre initially demonstrates elastic deformation which is a contribution from the organic material, before transitioning to inelastic deformation as the nanoasperities and interlocks are forced past one another (Figure 6). In compression, there is at first elastic deformation as the organic layers are squeezed to their full extent before it seems the platelets are unrecoverably forced apart. Finally, under tension the nacre behaves inelastically as dilation bands form and the platelets are pulled apart.

**Figure 7:**
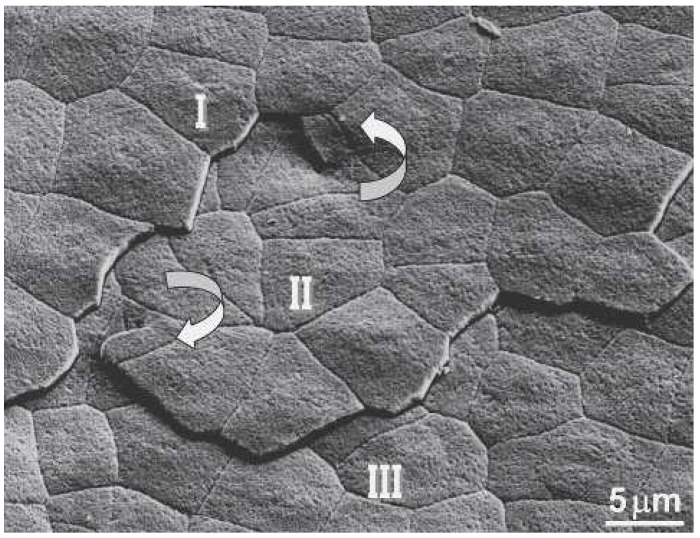
SEM image showing two screw dislocations and spiral growth associated with three layers in nacre. The center core and corresponding spiral growth domain are oriented counterclockwise connecting layer I and II; the core and corresponding domain at bottom left are clockwise relating to layer II and III. This shows how the layers are forming simultaneously on top of one another. Reproduced from [1] with permission from Cambridge University Press.

To further investigate the benefits of this phenomenon, Yao *et al.* used a nanomanipulator to remove individual platelets surrounding the singularity and followed it down through several layers. As they moved downward, they found that the center shifted slightly in each which consequently offsets the platelets and their boundaries (Figure 7) [1]. From this they concluded that the way in which nacre grows improves its fracture resistance by limiting crack propagation pathways, dislocation strengthening, and interlocking dislocation cores [1].

While nacre contains both an organic and an inorganic phase that work together to produce the best results, Barthelat and Zhu have produced a larger model for the system with only the inorganic phase represented in order to understand its contributions to mechanics [19]. Their goal was to understand how nacre spreads strain across such a large area and how this can be replicated in biomimetic materials. Their model system consists of micron-sized poly-methylmethacrylate tablets held together by fasteners that represent the surface effects of abutting tablet surfaces [19]. The tablets are waved to duplicate the “dovetailing” found in nacre and avoid the localization of stress that would occur with flat tablets [19]. Their system allowed them to easily measure the effect of tablet angle, preload on the fastener, and “unzipping” that occurs due to non-uniform deformation [19]. To more fully represent the mechanics of nacre, they would need to also then include a viscoelastic matrix phase that could deform as needed and lubricate the tablet interactions. Computer models made by Zhang *et al.* have shown that water molecules actually affect nacre most by altering the protein-mineral interactions at grain boundaries [20]. Hydration increases the fracture toughness of nacre by covering the mineral surface and forming hydrogen bonds with the protein [20]. Shear at the interface between the tablet and the matrix will deform the matrix until it fails and the tablets begin to slide past one another. This dissipates energy as the area transitions from the elastic to inelastic deformation regime. In the case of tension, the organic layer contains many sacrificial van der Waals associations which can be pulled apart to dissipate energy in a sawtooth pattern [11,20]. All these factors combine to yield one of nature’s most impressive materials; however, there are other biomaterials which derive their strength from nanostructure and hierarchical organization.

### 2.2. Hydroxyapatite and Bone

While nacre is one source of ideas for creating stronger nanomaterials, other researchers have looked to the human body for ideas. While materials implanted into the body are more frequently formed on the macroscale, they need to have nanoscaled features and structure to adequately replace those produced naturally. While the organization of bone is different from nacre on the macro scale, they are very similar in that their mechanical properties depend strongly on hierarchical organization over many lengthscales. Similar to nacre, bone is also composed of a strong inorganic component surrounded by a more ductile organic matrix. Natural bones are made up of collagen fibrils with embedded carbonated apatite nanocrystals [21]. The fibrils are generally 80–100 nm in diameter and the apatite crystals measure 25 nm × 50 nm [22]. Adjacent collagen molecules form covalent cross-links and are structured such that gap regions remain open to serve as nucleation sites for the inorganic phase, and cause the final structure again to resemble a “brick-and-mortar” structure [23]. These fibrils are then oriented with respect to each other to further increase the mechanical properties of the bone and provide the desired function at each location [22]. Parallel arrays of fibers offer the strongest form of bone with an elastic modulus of 26 GPa parallel to the fibers and 11 GPa in every other direction [22].

Bone can also be organized as woven fiber bundles of up to 30 µm diameter which are in fact highly disordered but can mineralize especially quickly for embryonic growth or growth after fracture [22]. Mineral crystals in bone are also similar to nacre in that they maintain a preferential orientation which distributes applied loads such that they are shared between the inorganic and organic components [24]. On the nanoscale, the interaction between the two components is organized so that contact area is maximized. This increases the interfacial and fracture strength of the composite without altering the materials used [24]. Bones also take advantage of the fact that the body keeps them well-hydrated and collagen fibrils can rely on structural water to share the load on bones [25]. About 10% of a bone consists of water and this water does not affect the structure of the bone; instead it binds very tightly to the fibrils and forms hydrogen bonds among its closest neighbors [23,25]. It therefore acts as a plasticizer and weakens the surrounding chemical bonds to lubricate the protein [25]. Lastly, these hydrogen bonds can break easily and then reform in a new orientation, allowing them to dissipate energy like the lustrinA in nacre [25].

Numerous attempts have been made to model this staggered structure in order to glean a better understanding of the hierarchy of bone and how it handles stresses. These same models can also then be run with conditions that mimic natural hard biological materials with and without water in order to determine the importance of hydration. Dubey and Tomar started with a tropocollagen organic phase and a hydroxyapatite inorganic phase which they modeled in a nanoscale staggered arrangement [26]. Then they performed molecular dynamics simulations in a chemical environment that was either non-hydrated, hydrated, or hydrated with calcium ions. They found that both the presence of water and calcium ions causes the Young’s modulus, ultimate strength, and toughness of hard biological tissues to increase [26]. This is because the water has a stabilizing effect on the collagen triple helix and also makes strong hydrogen bonds between the organic and inorganic phases. Another more generic model for biocomposites was developed by Bar-On and Wagner who staggered stiff platelets within a soft matrix and then applied the model to a collagen fibril [27]. They found that deformation governs displacement and that a shear-lag model can correctly predict material properties such as the effective modulus [27]. Dutta *et al*. used another model to calculate critical overlap length of the inorganic particles in an organic matrix [13]. They found that shear stress will be maximized at both ends of the overlap and that natural overlap lengths are optimized to decrease shear stress such that it is equivalent to the shear strength of the organic matrix [13]. Begley *et al*. looked at the mechanical properties as a function of aspect ratio in brick-and-mortar composites [28]. They found that for shorter crystals the failure of the vertical interface controls the strength but as the length grows the strength is more governed by pull-out stress [28]. They go on to note that for the strongest mortar there will be only a narrow range of acceptable brick sizes for which the pull-out stress is dominant [28]. More work by Dubey and Tomar found that the water also acts as a lubricant between tropocollagen molecules during tension but as a glue during shear [29]. This lubricating effect led to a reduction in compressive strength but the trade-off to the system is that the strength is increased in tension [29].

**Figure 8:**
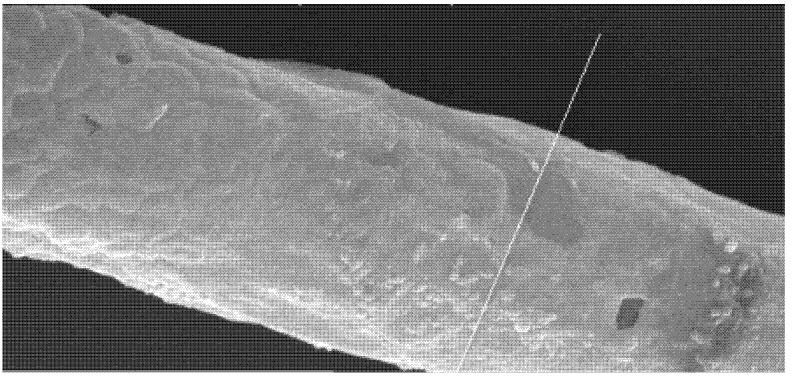
High-magnification SEM photograph of hydroxyapatite fibers. Reproduced from [31] with permission from John Wiley and Sons.

For tissue engineering purposes, it is desirable that fibers with similar mechanical and chemical properties be easily produced and processed to fit the intended function (Figure 8). Such fibers are often made by electrospinning techniques which can yield fibers with diameters less than 100 nm [30].

Similar fibers can also be achieved by sol-gel electrospinning, but with the added disadvantages that the material requires additives to get the necessary viscosity for spinning and a high temperature calcination step is required to remove them. Rather than wait for an apatite layer to form, these concerns can be eliminated by instead using newly developed electrospinning methods which produce hydroxyapatite fibers from the start. These engineered hydroxyapatite fibers can also benefit from the addition of helpful nanoparticles that can stiffen the matrix and contribute to the chemical properties of the structure without detracting from the mechanical strength. As such, the possibilities for material improvement are extensive and could be used to address a variety of situations.

### 2.3. Bioactive Glass Nanoparticles

**Figure 9:**
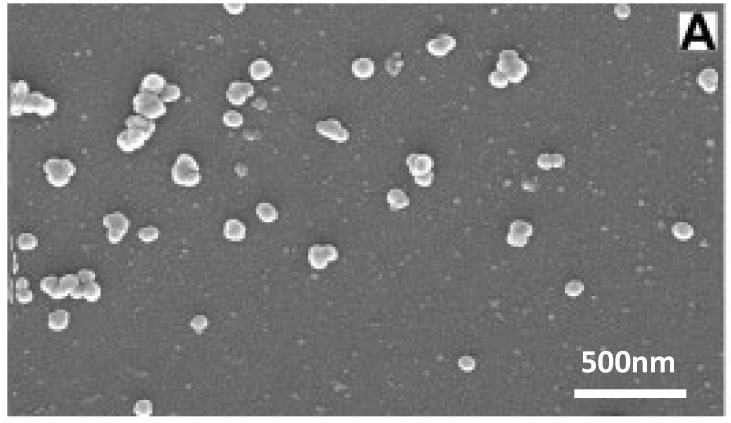
SEM of spherical bioactive glass nanoparticles with the formulation SiO:P_2_O_5_:CaO = 55:40:5 (mol). This demonstrates the homogeneity of the nanoparticles and also the aggregation characteristics. Reproduced from [32] with permission from Elsevier.

Another fascinating material worth exploring is the useful ceramic, Bioglass. This is the commercial name for one of several glass-ceramic materials which are often used in tissue engineering due to their favorable mechanical properties and high bioactivity. When these glasses come into contact with physiological fluids (or simulated body fluids, SBF) their surface reacts to form an apatite layer which allows the material to form strong bonds to bone [30]. This means that their applications can range from tissue scaffolds to surface coatings to filler material in composites. Initially, research into their properties was done with micrometer-scale particles, but Boccaccini *et al*. found substantial improvements in biological function by transitioning to nanoparticles instead (Figure 9) [30]. The switch to the nanoscale means that the particles have a higher surface area to volume ratio which leads to faster release of ions, deposition, and tissue mineralization as well as higher protein adsorption [30]. Nanoparticles also result in a better imitation of bone which naturally has nanosized hydroxyapatite particles, and therefore cells and proteins can adhere to nano-features. Boccaccini also points out that the reduced size helps them to stiffen polymeric nanofibers without causing structural disruption, to be processed into thin bioactive coatings, or used as an injectable system [30].

**Figure 10:**
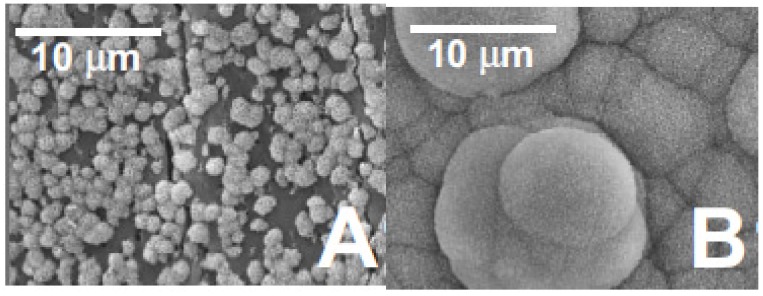
SEM images of different substrates after immersion in SBF for two weeks at (**A**) 25 °C and (**B**) 37 °C. Set 1 corresponds to PNIPAA-grafted PLLA films with 20% bioactive glass particles. The thermoresponsiveness of the particles is demonstrated by the selective growth of apatite after increasing the temperature. Reproduced from [34] with permission from John Wiley and Sons.

Some additional improvements to these particles can come from surface coatings or other processing techniques that prevent agglomeration and help integrate the Bioglass into polymer matrices to produce superior composite materials. However, Liu *et al*. found that the composites containing surface-modified nanoparticles had greater tensile strength than unmodified nanoparticles [33]. The surface-modified composites also exhibited higher nucleation and crystallization rates because the nanoparticles acted as nucleating agents and the surface-modified particles were more uniformly dispersed in the scaffold. Finally, by using a mask during plasma activation, it is possible to selectively functionalize only part of the polymer surface so that apatite growth can be triggered only in predetermined areas [34]. Shi *et al*. concludes that this patterning ability especially could be used for the differentiation and control of cell growth on implant surfaces (Figure 10) [34].

Bioglass and other ceramic glasses are not the only nanoparticles that have contributed to the improvement of materials in the body. Lee *et al*. have found computationally that nanoparticles dispersed in a polymer film can actually preferentially deposit themselves at sites where they are needed [35]. They are driven there by the polymer chains in the system which will gain conformational entropy by stretching to accommodate particles within the film [35]. This causes aggregation of the nanoparticles and pushes them into cracks in the surrounding matrix [35]. Once they are in place, and if there is a continuous path bridging the crack surface, the nanoparticles are capable of transferring loads away from the cracked matrix to themselves [35]. These trapped particles then bind to the matrix and, if they are bioactive, begin to heal it. Due to their small size, these particles can easily be incorporated into a variety of scaffolds, injected, or administered to the site of interest by other means. However, bioactive glass-ceramics are not the only innovation in nanoparticles for the life sciences, there are also a variety of polymeric nanoparticles.

### 2.4. Drug Delivery

Nanomaterials have made a monumental impact on the field of drug delivery. Unlike larger particles, nanoparticles have the ability to reach far more sites in the body, can circulate for longer periods of time, have higher effective surface areas, can control drug release, and can even cross the blood-brain barrier. The incorporation of drugs into nanoparticles allows them to be delivered directly to the site where they are needed, which is more efficient and reduces the required dosage. Nanoparticles are also capable of passing through Peyer’s patches which regulate the environment of the small intestine and therefore can be administered intravenously [36]. However, nanoparticles also face challenges such as undesirable recognition as a foreign body and consequent filtration by the liver or spleen before the drug can be delivered. To avoid this, there are several broad solutions which must be optimized for each drug-nanoparticle combination to obtain the intended size, release rate, and circulation time.

Before the nanoparticles can deliver a drug to its target, the drug must first be incorporated into the particle. While the drug could theoretically be attached to the nanoparticle after formation via adsorption, larger amounts can be incorporated by introducing the drug during the nanoparticle formation process [37]. Common methods for this step which will be presented briefly include: solvent evaporation, spontaneous emulsification, salting out, supercritical fluids, coacervation, and polymerization. Solvent evaporation refers to a process in which the desired drug and polymer are dissolved in an organic solvent, emulsified with an aqueous solution, and then solvent is evaporated from the emulsion [38]. However, this process is energetically expensive and does a poor job of encapsulating hydrophilic drugs [39]. Spontaneous emulsification is the same as solvent evaporation except a water-soluble solvent is also added to increase diffusion in the emulsion and therefore reduce the resulting particle size [37]. The spontaneous method is also faster and results in a more porous product than that achieved by evaporation [21]. Salting out was developed in order to avoid using chlorinated solvents which leave behind harmful residuals that can be difficult to remove and degrade components that they come in contact with. In this process, the addition of a salting-out agent allows a liquid-liquid phase system of normally miscible compounds to form. This forms an oil-in-water emulsion to which excessive amounts of water are added to yield the nanoparticles [22]. The supercritical fluid method also aims to get around using harmful solvents in the production of nanoparticles. It can be done in two ways. The first is involves the rapid expansion of a supercritical solution such that the dissolved solute precipitates out when the solution is expanded and loses its improved dissolving power [37]. However, because very high molecular weight polymers are preferred for many drug delivery applications and they have low solubility even in supercritical fluids, production has shifted to a supercritical anti-solvent method [37]. In this method, the solute is dissolved in an organic solvent and precipitates as the pressure is increased to the point that all residual will be removed [37]. Coacervation also uses phase separation to create nanoparticles. Polymerization methods depend on the polymer chosen for the application; however, generally the drug is dissolved in an acidic polymerization medium before the addition of the monomer and then the polymer forms by anionic mechanism with mechanical agitation [31]. To improve the quality of the product and prevent nanoparticle aggregation, surfactants and stabilizers can be added.

Next the nanoparticles need to be optimized for their intended function. Important things to consider include the final size of the particle, aggregation phenomena, and surface characterization. Encapsulation efficiency is known to correlate with the diameter of the nanoparticles so rapid dissolution can be achieved with particles on the order of 100 nm and more sustained dissolution with particles of approximately 800 nm [39]. At the same time, the target site of the nanoparticles is an important consideration. For example, nanoparticles whose surfaces had been modified with chitosan were found to have increased penetration to mucosal surfaces [39]. Nanoparticles in the body also have to contend with plasma protein adsorption, phagocytosis, and the MPS [40]. Phagocytes will attach to the nanoparticle surface when opsonins in the blood adsorb onto the hydrophobic portions of the nanoparticle, but this can be prevented by grafting hydrophilic particles such as polyethylene glycol onto the surface [40]. While the activity of phagocytes may be helpful if the intended target is in fact the liver, this uptake could be dangerous if there are cytotoxic components in the nanoparticles [40]. Initially, researchers considered solving this problem by suppressing the reticuloendothelial system; however, this could lead to a new set of problems and therefore has not been an ongoing focus of research [40]. Rather, hydrophilic nanoparticles are favored for drug delivery applications because they are filtered out at a much lower rate than their hydrophobic counterparts. To take advantage of this fact, nanoparticles can either be polymerized from hydrophilic polymers to begin with or treated after production to become so [37]. Hydrophilic polymers frequently used in polymerization include chitosan and gelatin, which can both form nanoparticles through an ionic gelation method [37].

## 3. Applications

The applications for these innovations are many and the work so far can be divided into three broad categories or “generations”. Hench and Polak describe how the first generation of biomaterials was designed to be inert in the body; to serve their mechanical function without negatively impacting the surrounding tissue or being enveloped in a fibrous capsule [41]. However, even materials that weren’t cytotoxic were still rejected by the body or produced microscopic debris, so surrounding tissues would develop a barrier to encase the implant and keep it separated. The second generation of biomaterials looked to get around this by developing materials that were bioresorbable or bioactive. The bioresorbable materials were designed to be implanted in the body and then slowly be degraded into harmless by-products as the living tissues regenerated and resumed their roles in the body [41]. On the other hand, more permanent solutions were found with the implantation of devices with bioactive properties or bioactive coatings. The most notable examples of this are various hydroxyapatite coatings which mimic the structure of natural bone and can provide strong adhesion between devices and hard tissues. These devices could still perform the mechanical tasks of the first generation but would not be rejected by the body. Finally, the third generation is attempting to go one step further and elicit reactions from the surrounding cells. So not only will the device perform its mechanical function and be accepted by the body, but it can also stimulate the surrounding cells to focus their efforts towards repair by activating genes, directing the growth of osteoblasts, or releasing growth proteins during the degradation process [41].

### 3.1. Biomimetic Nacre and Layer-by-Layer Processing

The applications of nacre have less to do with the material itself and much more to do with the implications of its structure for man-made materials. While nacre has improved mechanical properties that arise from its micro- and nanostructure such as nanoasperities, the sandwiching of the organic layer, and platelet interlock mechanisms, their mere existence does not provide enough information to duplicate them in engineered products. For this reason, there is also a great deal of research dedicated to the manner in which nacre forms. Biomedical devices today are often detailed combinations of processing methods designed to produce the best interactions with the body. For example, artificial nacre-like coatings have been fashioned by a lamination process which is referred to as a layer-by-layer approach. Several different methods have been developed to produce thin layers that structure themselves so as to enhance the mechanical behavior of the individual components.

One layer-by-layer approach was used by Tang *et al*. to produce nanostructured artificial nacre of polyelectrolyte multilayers which mimicked the tensile strength and organic matrix of nacre [42]. They layered montmorillonite clay tablets with polyelectrolytes such that the tablets oriented themselves parallel to the surface in order to maximize attractive energy [42]. The result was a composite of films measuring about 50–200 nm where each individual clay/polyelectrolyte layer is about 3 nm thick, and the polyelectrolyte layers closely mimic the function of those found in nacre [42]. Over 75% of the molecules are tightly coiled such that they contain similar sacrificial loops which help dissipate energy, prevent deformation, and strongly adhere to the clay surfaces [42]. Tang *et al*. measured a tensile strength of up to 100 MPa and a Young’s modulus of up to 11 GPa, and explained the surprisingly low Young’s modulus by noting that the montmorillonite platelets lack the nanoasperities found in nacre which Katti proved provide additional friction between layers [42]. Lin *et al*. also used montmorillonite as their stiff component but used a combination of hydrothermal and electrophoretic assembly to intercalate polymer into the montmorillonite [43]. They found that the driving force behind the laminate’s structure was the increase in entropy caused by desorption of solvent molecules and therefore the process relies on self-assembly [43]. However, the layers in their composite are much thinner than those found in natural nacre and therefore there was less space for polymer folding and cross-linking which provides nacre’s organic layer with many of its characteristic deformation properties [43].

A similar layer-by-layer approach was used by Wei *et al.* to produce polymer thin films that closely mimic the organic and inorganic components of nacre [44]. They emphasize the importance of the insoluble biomacromolecules that act as a framework as well as soluble biomacromolecules which offer negatively charged surfaces for the nucleation of aragonite platelets [44]. To mimic this in their film, they alternated diazo-resins and poly(acrylic acid) to provide the framework and nucleation sites respectively, and then treated these organic multilayers with CO_2_ gas diffusion which was tuned to provide the desired CaCO_3_ thickness [44]. The result was a film that closely mimics the structure of nacre and a model for the adjustable fabrication of layer-by-layer nanocomposites. This method results in thinner, more homogenous, and less ordered layers but does not allow for coating complex geometries [3,45].

Researchers have used their knowledge of the structure of nacre to produce biomimetic materials that are stronger than the composite materials would be separately. From nacre they have gleaned that flaw-intolerant inorganic ceramics are strong but the ductile organic polymers which surround them are weak [46]. Therefore they want to combine the two in order to access only the advantages of each. Surprisingly, some groups have found that they actually do not need the high volume fraction of inorganic material found in nature to achieve substantial improvements [46]. In fact, one lamination process proved that composites with platelet concentrations higher than 10% vol were more brittle than their sparser counterparts and did not offer a compensating increase in strength [46]. Bonderer proposes that since nature is restricted to relatively brittle ceramic components, these must be present in high quantities to provide the necessary strength for the material [46]. However, material scientists are not similarly restricted and Bonderer proved that a layer-by-layer design of alumina platelets and chitosan matrix could form a laminate with an elastic moduli equivalent to that of bone [46]. The importance of the relative fractions of the two components was also investigated by Sun *et al*., who layered chitosan and hydroxyapatite to test the relationship between the composition ratio, and the homogeneity and mechanical strength of the composites [47]. They found that composition was most likely related to the degree of microscale-aligned stacking of cross-linked layers, and how improves tensile strength and flexibility [47]. However, this system was arduously assembled by forming a polymeric foam via a sublimation-drying process and then compressing the foam into a nacre-like structure [47]. Yao *et al.* proved that the nacre’s same organic matrix and inorganic tablet system could be achieved using self-assembly along with layer-by-layer process to attain the same mechanical improvement [48]. They layered chitosan molecules onto montmorillonite nanosheets and then induced self-assembly by vacuum-filtration or water-evaporation to force the chitosan to form electrostatic and hydrogen bonds to the substrate [48]. In doing so, the material’s surface roughens such that nacre-like surface features were present and they saw 2–5 fold improvement in the Young’s modulus and ultimate tensile strength compared to films without this self-assembly step thereby demonstrating the importance of the nanostructure in these materials [48].

Another interesting system that combines an inelastic and an elastic component is “tough hydrogels” which are formed from ionically and covalently crosslinked polymer networks. These have been compared to the organic matrix found in nacre due to their similar viscoelastic properties. Hydrogels have been used as scaffolds and drug delivery vehicles already, but very durable models are currently being developed by Sun *et al*., which are notch-insensitive and can remember their original state [49]. The ionic network absorbs energy as bonds break easily across large areas and the covalent network maintains structural integrity by crack-bridging. Systems such as these are very promising for fields such as tissue engineering because of their ability to be seeded with cells and then implanted directly into the human body.

Both of the above examples of composite materials can also contain small additives to improve device performance. One example of this is increased antibacterial activity, which can be achieved by the inclusion of silver nanoparticles. Silver has long been known to have antibacterial and anti-inflammatory activity; however, Lok *et al*. proved the antibacterial activity is related to size with the smaller particles having higher activity [45,50]. They also found that by stabilizing the surface with albumin they could prevent aggregation of the nanoparticles. Aggregation is best avoided because it results in decreased effective surface area as well as limits the degree with which they can associate with bacterial cells [50]. However, there are also concerns that releasing large amounts of silver into the human body can be detrimental and so Podsiadlo *et al*. produced layer-by-layer composites with immobilized silver nanoparticles that could not enter the body [45]. They used an assembly of poly(diallylmethylammonium chloride), montmorillonite clay, and silver nanoparticles coated with starch to prevent aggregation [45]. As a result of the immobilization of the silver within the film, Podsiadlo *et al*. was able limit the amount of eluted silver to less than 3 µg/L which is not detrimental to mammalian tissue [45].

### 3.2. Nanostructured Scaffolds

**Figure 11:**
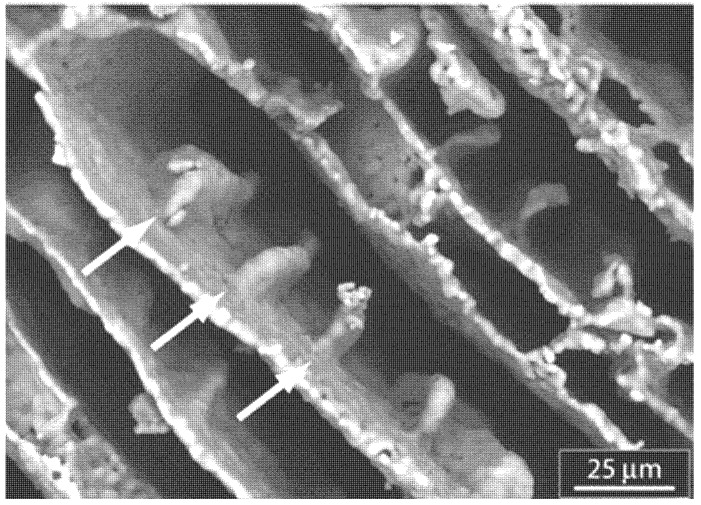
Surface dendrites, oriented along the ice growth direction, cross section perpendicular to ice growth direction. These may guide cell growth as they penetrate the structure. Reproduced from [51] with permission from Elsevier.

These layer-by-layer composites and hydrogels are wonderful for integration with the body, but there is also strong interest in scaffolds containing other nanocomponents. One of the cleverest ways that researchers have found to produce these scaffolds is by freeze-casting. To form its desired crystalline structure, a water-based slurry will eject any solutes into channels between the ice crystals. If the included solute is a ceramic, setting the material and then draining the water will leave a scaffold that makes a very dense composite [3]. While that is a good demonstration of the premise, researchers have recently discovered ways to do the same with hydroxyapatite. Before freeze-casting was developed, porous scaffolds made from hydroxyapatite were too weak to bear loads. One common method was the particulate leaching technique used porogens, gelatin spheres or salt crystals, to give the scaffold porosity and could later be leached out of the foam with water to leave the desired vacancies [1]. These left pores of about 300 µm which is desirable for osteoblast conduction but the same technique can be applied with nanoparticles to yield highly porous foams with high interconnectivity of pores. Moreover, since then, freeze-casting has allowed for the production of hydroxyapatite scaffolds with unusually high compressive strength [51]. Deville *et al.* used a technique based on a ceramic slurry that is poured into a mold, then freeze dried and sublimated under pressure (Figure 11) [51]. They were able to produce foams with lamellar, cellular, or dense microstructures depending on the processing conditions. The strength of the lamellar regions was similar to that of compact bone with a compressive strength of 145 MPa for foams with 47% porosity and 65 MPa for 56% porosity [51]. In this experiment, Deville *et al*. used initial hydroxyapatite particles which were about 2.4 µm in size, however they noted that repelling small particles from the supercooled ice front is easier than repelling larger particle and even smaller particles have the potential to reach lower porosity without sacrificing the interconnectivity of the pores.

Of particular interest in the freeze-cast system are nanoscaled surface dendrites found within the pores. These reoriented along the ice growth direction and could potentially serve as guidance for cell growth as it infiltrates the scaffold [51]. A different method was used by Fu *et al.*, who was able to produce dense scaffolds with a unidirectional pore structure by directional freezing of aqueous hydroxyapatite suspensions on a cold substrate [52]. These scaffolds could then be sintered to increase the material density without damaging the microstructure. While this work did not extend to testing these scaffolds in the body, he posits that they would be good for bone engineering applications because the initial aqueous phase could be mixed with glycerol or dioxane to achieve a high degree of control over the final microstructure [52].

Another ice-templated system was attempted by Launey *et al*., who created a ceramic suspension to serve as the matrix which was then filled with metal to form tablets [53]. In order to successfully model the complexity of many length scales found in a natural system, they added sucrose to give the final structure microscopic asperities and designed their system to mechanically align the lamellae [53]. While they chose sucrose for their system, they also noted that other additives could be used in order to maximize the interfacial tension, surface roughness, degree of supercooling, and viscosity [53]. This system could even be further improved by grafting a methacrylate group onto the ceramic before the metal infiltration in order to form covalent bonds between the two components [54]. The result was a composite which did not demonstrate failure by delamination and showed crack-bridging ligaments, both of which testify to the beneficial mechanical properties of the matrix [53]. The material could support tensile strains greater than 1%, toughened during crack propagation, and had a fracture toughness twice that of the bulk materials [54].

Ahn *et al*. also found that the presence of yttria-stabilized zirconia nanocrystals can stabilize the homogeneity of the hydroxyapatite matrix, which is inherently prone to phase impurities and heterogeneity, to induce fracture toughness equal to that of bone, and to prevent decomposition at high temperatures [39]. Even hydroxyapatite nanoparticles can be implanted in scaffolds. Dong *et al*. produced polyurethane foams to which nano-hydroxyapatite was added to produce a non-cytotoxic and degradable scaffold for joint engineering [55]. Polyurethane is commonly used in biomedical applications for it mechanical properties, biocompatibility, and resistance to fatigue. The result was a scaffold with 80% porosity, pores ranging from 100 to 800 µm, and a compressive strength of approximately 271 kPa [55]. The scaffold also integrated well with the body; it was fully covered by cell growth within 7 days in SBF and noticeably degraded 12 weeks after implantation [55].

Polymer matrices also offer novel combinations of nanoparticles that have advanced the field of biomimietics. Bones in the body have an impressive ability to self-heal that researchers would be thrilled to replicate or improve upon. The Wolff-Roux law describes how bone can adjust to its environment over a period of 4–10 years by removing material from some sites and then depositing more where it is needed [23]. If an area of bone is damaged, the body can send osteoclasts in to remove the failing cells. Then, osteoblasts on the site will fill in the area such that the area is replaced with fresh bone until the osteocytes on the surface of the bone register uniform local loading [8,23]. This has been duplicated in polymeric matrices by including capsules of resin which contain a healing agent and dispersing catalytic nanoparticles throughout the material [21]. When a propagating crack reaches the healing agent, it spills out until it reaches a catalyst and seals the gap [21]. By including nanoparticles in the bulk material, only a small amount of catalyst is needed to ensure that any crack will encounter the healing agent before it propagates too far. However, the microcapsule that held the healing agent will remain behind and can disrupt the fiber architecture. Another method in the same vein consists of creating complementary networks of tubes throughout the matrix that carry the healing agent and catalyst [56]. This method can provide a higher volume of healing agent to the damaged site and the fibers can be oriented to match the nanostructure of the polymer matrix [56]. Trask also mentions work by Verberg *et al*., who have encased nanoparticles in a microcapsule such that they can diffuse out and therefore travel through channels repairing damage as they go along [56].

While polymeric foams can be reinforced with helpful nanoparticles, they can also benefit from the inclusion of short hydroxyapatite nanofibers. In fact, there is research that suggests that complete fibers offer more benefit than particles [57]. These fibers can be made by a procedure involving a liquid phase synthesis with a metal-organic precursor which is then converted to the desired inorganic material via electrospinning [30]. A better formation technique is laser-spinning which can quickly make fibers with diameters of 200–300 nm that can form an apatite layer in SBF within 5 days [30]. This process differs from the first in that a small amount of material is superheated by a laser and then blown out with an injection of supersonic gas that stretches and cools the material [30]. In all cases, the fibers can be collected as a mat which will contain pores of sufficient size for osteoblast infiltration, about 300 µm, and can be used effectively for non-loadbearing bone engineering. One particular technique perfected by Hench can produce smooth fibers with a 50 µm diameter [31]. However, once the fiber was calcined to remove the polymer and leave pure HA, the diameter dropped to 10–30 µm and 1 µm diameter HA grains could be distinguished [31]. This calcined fiber gives rise to nanoscale features which much better imitate those found naturally in the body and encourage the growth of cells on the surface. Bulkier structures than fibers such as nanostructured composites can be made by chemical precipitation and variables such as the pH, crystal size, and surface chemistry allow for control of the final nanomorphology and composition [39].

**Figure 12:**
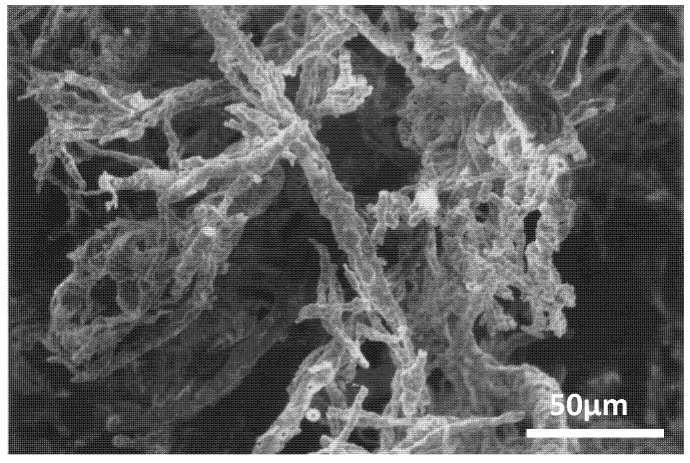
SEM photomicrograph of hydroxyapatite fibers incorporated into a polymer foam matrix. Reproduced from [57] with permission from Elsevier.

Some of the first work regarding fibers incorporated into matrices was done by Thomson *et al*., who found that the addition of micron-sized fibers could reinforce low-porosity poly(DL-lactic-co-glycolic acid) foams for enhanced osteoconductivity and compressive yield strength (Figure 12) [57].

**Figure 13:**
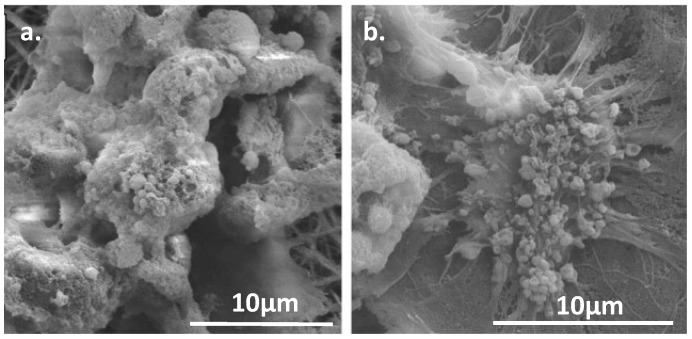
(**a**) Mineral depositions on the electrospun nanofibrous scaffolds: apatite-like morphology of deposit at higher magnification, (**b**) Globular minerals and collagen bundles associated with a single cell viewed at higher magnification. Reproduced from [58] with permission from Elsevier.

PGLA was chosen for several reasons; it can be seeded with osteoblasts for improved proliferation, is bioresorbable, and is FDA-approved. However, in its natural state it does not have the compressive strength necessary for load-bearing applications. Hydroxyapatite can offer this increase in compressive strength and is also a resorbable material in addition to being osteoconductive. Thomson’s group was able to produce foams with a porosity of up to 0.47 and compressive yield strengths to a maximum of 2.82 MPa whereas the foam without fibers had a compressive yield strength of only 1.35 MPa [57]. However, groups have found that using nanofibers offers even more benefits. Zhang *et al*. created composite nanofibers of hydroxyapatite and chitosan which could then be incorporated into chitosan scaffolds for improved function in the body (Figure 13) [58]. With the addition of ultrahigh molecular weight poly(ethylene oxide), spindle-like hydroxyapatite nanoparticles and chitosan fibers could be electrospun into nanofibers which successfully promoted the growth of apatite and cells on the scaffold after submersion in SBF [58].

### 3.3. Bioactive Glass Nanoparticles

As mentioned before, bioactive glass nanoparticles are highly desirable for biomedical applications because they will react with SBF to form an apatite layer. As it does so, the reactions taking place at the surface of the particle will cause it to release dissolution products into the system. These solutes can be tailored to best help the cells to adhere by up-regulating the expression of genes that control osteogenesis, releasing desired ions, or secreting vascular endothelial growth factor [30]. To improve the function of bioactive glass nanoparticles in composites, researchers have paired them with favorable bioresorbable polymer matrices. Examples of this include poly(3hydroxybutyrate) for superior water absorption, poly(L-lactic acid) which can be produced by a faster sol-gel process, and polysaccharides naturally found in the body such as chitin, collagen, or chitosan [30]. To improve the integration of the nanoparticles with the matrix, organic molecules have been grafted onto the surface of the nanoparticles to form better bonds and also help keep the nanoparticles dispersed [30].

**Figure 14:**
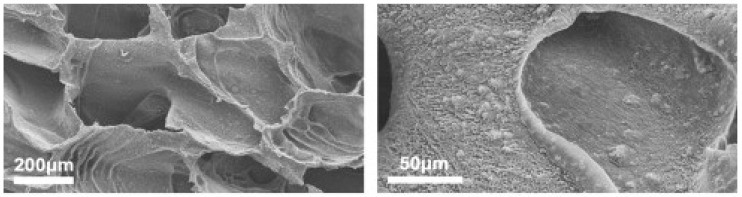
SEM morphology for the porous PLLA/BGC scaffolds with 20 wt.% BGC content: at low-magnification; at high-magnification 20 wt.%. The interconnected pores and nanoparticles studding the surface are visible. Reproduced from [32] with permission from Elsevier.

Scaffolds also benefit enormously from the incorporation of bioactive glass ceramic nanoparticles. One example of this are the poly(L-lactic acid) scaffolds created by Hong *et al.* (Figure 14) [59]. They used a thermally induced phase-separation method to introduce several different weight percents of nanoparticles and then observed their impact on the porosity, compressive strength, and compressive modulus of the foam. The particular particles they chose were composed of SiO_2_, CaO, and P_2_O_5_ in a molar ratio of 55:40:5. They chose to make them nanosized (20–40 nm) because the larger specific surface area allows for a tighter interface with the polymer matrix and improved biomineralization [59].

**Figure 15:**
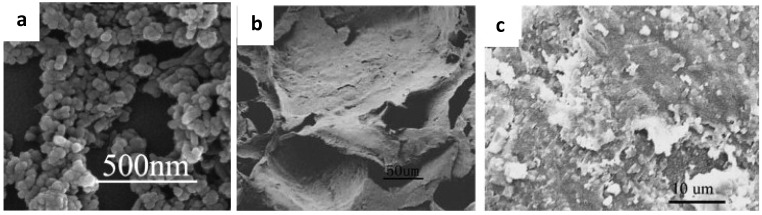
(**a**) ESEM micrograph of bioactive glass nanoparticles sintered for 3 h at 600 °C; (**b**) The scaffold morphology: PLLA for 7 days; (**c**) g-bioactive glass/PLLA composite for 7 days. Reproduced from [33] with permission from Elsevier.

Their optimal foam was achieved by including 20 wt.% of nanoparticles which resulted in highly interconnected 20–400 um pores, a compressive strength of up to 0.35 MPa, and a compressive modulus of 8 MPa [59]. These nanoparticles not only improved the mechanical properties of the foam, they also contributed to its bioactivity. The 20 wt.% foam nucleated apatite over its entire surface within a day whereas EDX performed on the neat foam showed no nucleation after three weeks of immersion in SBF [59]. One example of this is work by Liu *et al*., who modified the surface of their bioactive glass nanoparticles by coupling them to poly (L-lactide) with diisocyanate (Figure 15) [33].

They found that the PLLA composites failed by ductile fracture and those containing bioactive glass nanoparticles had a lower tensile strength then pure PLLA. While Liu *et al*. initially mention concerns about the toxicity of isocyanante, they found that their scaffolds developed a complete coating of apatite after being submerged in SBF for 7 days and seemed to have good biocompatibility [33]. Shi *et al.* also altered the surface of bioactive glass nanoparticles but the result was a “smart” surface that would preferentially form apatite in patterns under specific stimuli [34]. They did this by first synthesizing poly-(N-isopropylacrylamide) onto a film surface that had been plasma activated. This polymer was chosen for its thermoresponsive properties; at approximately 32 °C it changes from a hydrophilic to hydrophobic state which causes a shift in conformation [34]. These altered nanoparticles were mixed with poly (L-lactic acid) at 20 wt.% and submerged in SBF for two weeks at 25 °C [34]. At the end of the two weeks, there had been no apatite formation; however, raising the temperature to 37 °C resulted in the formation of apatite aggregates [34]. This has been successfully attempted with other foams as well. Roether created poly(DL-lactide) (PDLLA) foams with macro-pores (>100 nm) and nano-pores (20–30 nm) which are oriented along one axis due to the nature of the cooling process [60]. The macro-pores are large enough to allow osteoclasts to attach and infiltrate the foam which assists the scaffold with its integration into the body while the nanopores offer extremely high surface area for the adhesion of proteins and hydroxyapatite. PDLLA foams are also desirable because they will slowly degrade without forming any acidic byproducts that will cause inflammation in the body or crystalline masses that will remain lodged in place [60]. However, Roether *et al*. then went one step further and incorporated Bioglass nanoparticles by adding them to the composite and coating them onto the scaffold surface [60]. The result was a scaffold that formed hydroxyapatite crystals within a week and a continuous hydroxyapatite layer within three weeks, but also degraded as a function of time in contact with SBF [60]. Since Bioglass can form bonds to both hard and soft tissue, the addition of the nanoparticles resulted in a more versatile scaffold with improved bioactivity and the nanoscale particles did not interrupt the structure or impede osteoblast conduction.

**Figure 16:**
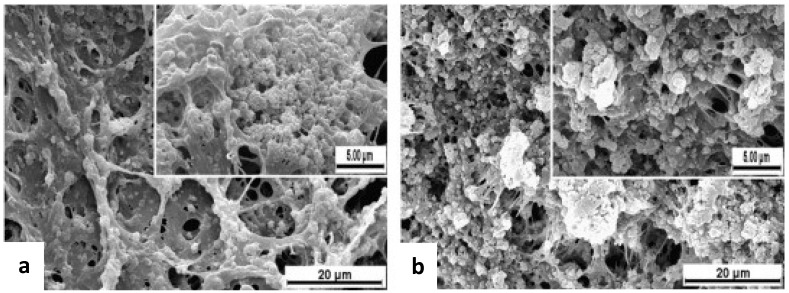
SEM images of hydrogels with different BG contents after being immersed in SBF for 14 days: (**a**) BG-40% and (**b**) BG-50%. Apatite has blossomed on the scaffold in the form of budding spheres. Reproduced from [32] with permission from Elsevier.

Bioactive glass nanoparticles have also led to innovative injectable solutions for bone engineering. Whereas a traditional scaffold must be formed outside the body and then seeded with cells before implantation, Couto *et al*. have developed a thermo-responsive hydrogel which can be mixed with cells and then injected directly to the damaged site (Figure 16) [32].

For their polymer matrix they have chosen chitosan because it is biodegradable, biocompatible, nontoxic, and derived from the widely available polysaccharide chitin. The only downside of chitosan is that it must be mixed with β-glycerophosphate salt in order to maintain a neutral pH, but the salt does not impact the chemical or mechanical function of the scaffold [59]. Previous injectable systems have failed because they consisted of ceramics which need high curing temperatures that would kill cells [32]. In this system, the chitosan was combined with bioactive glass nanoparticles between 40–100 nm in diameter in varying weight percentages. They found that the gelation temperature decreased proportionally to the increase in percent nanoparticles with the physiologically optimal gelation temperatures of 36.9 °C and 36.8 °C being achieved at nanoparticle weights of 40% and 50% respectively [32]. They attributed this to either the release of ions from the nanoparticle surface facilitating hydrophobic interactions between chitosan molecules or simply the addition of an elastic component to the viscoelastic matrix [32]. The resulting scaffold could gel within 5 min after injection and form apatite within 3 days, therefore making this system particularly appealing for repairing bone defects which traditional scaffolds may be unable to heal [32].

### 3.4. Nanoparticles for Drug Delivery

**Figure 17:**
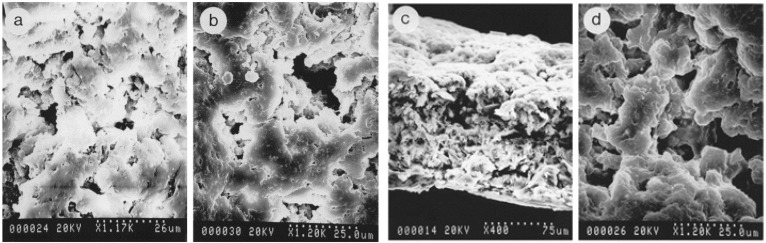
Scanning electron micrographs of the PLGA/F127 (90:10, *w*/*w*) matrices after 1-day (**a**: surface), 6-day (**b**: surface; **c**: cross section) and 18-day (**d**) incubations in buffer. These show the interconnected open pore structure and also that they extend to the surface without a decrease in size that would make them less accessible. Reproduced from [61] with permission from Elsevier.

Above we have discussed many novel ways to incorporate nanoparticles into scaffolds, but we can imagine one step further by considering loading drugs into these nanoparticles. First, it is very important to decide how the drug will leave the nanoparticle. The release rate of the drug will be dependent on desorption of any surface-bound drug, diffusion through the nanoparticle or capsule wall, and degradation of the matrix itself [38]. Most nanoparticles have an initial burst release of the drug adsorbed to the surface which has been found to be greater for smaller particles and for nanoparticles with greater drug loading [36]. Depending on the application, it may be preferable to speed up, slow down, or otherwise alter the rate at which the drug would be released from the nanoparticles. Work by Song *et al*. investigated the possibility of introducing additives to the initial mixture in order to achieve this (Figure 17) [61].

The initial nanoparticles were made of poly(lactic-co-glycolic acid) and demonstrate a biphasic release kinetics curve that with a slow diffusion release and then a fast degradation release phase [61]. Their goal was to produce a monophasic system that did not demonstrate the lag time found in the neat polymer. In each set of nanoparticles, they incorporated one of five additives with varying molecular size, hydrophilicity, and steric configuration. They found that the presence of hydrophilic molecules enhanced drug release from a hydrophobic matrix and that the water-soluble additives were leached from the matrix within the first three days [61]. Only one of their chosen additives, which had a high molecular weight and was water-soluble, resulted in the desired monophasic behavior. SEM analysis showed that this was because the additive created large interconnected pores connected to the nanoparticle surface which formed solvent-filled channels large enough for the drug to seep out [61].

Previously the criteria for successfully preparing nanoparticles loaded with a drug were broadly presented; however, there are many innovative direct applications that should be considered as well. One of the most challenging organs to deliver drugs to is the brain because it is protected by a blood-brain barrier which is composed of the basal membrane, and tightly packed endothelial and neuroglial cells. However, the brain is understandably also one of the most helpful targets for treating maladies from depression to tumors [62]. The brain has two main methods by which it allows molecules across the barrier. First, compounds that are at physiological pH, lipophilic, and have low molecular weight can pass through by diffusion [63]. Second, compounds that resemble specific proteins and other components needed for brain function have specified receptors in the blood-brain barrier which actively transport these [63]. The brain also has an impressive ability to keep things out. Junctions between cells are tight and circumferential such that there are no continuous pathways between them and pumps operating in the cell reject lipophilic compounds that should not be allowed to cross [32]. To get around this, nanoparticles are often coated or surface treated to produce more favorable interaction properties. Nanoparticles coated with polysorbate-80 and polysorbate-85 were found to effectively transport drugs through the barrier [32]. Later work by Schroeder *et al*., proposed that these coatings help bind nanoparticles to the lining of the capillaries such that they induce a large concentration gradient which facilitates diffusion or purposefully induce phagocytosis processes in the epithelial cells [64]. Kreuter found that nanoparticles coated with polysorbate-80 transported drugs through the barrier with a 20-fold improvement over the uncoated nanoparticles [62]. However, he concluded that it is because the polysorbate-80 coating attracts apolipoprotein E from the blood to the surface [62]. He believes that the nanoparticles then mimic low density lipoprotein to the extent that it can trick lipoprotein receptors into transporting it into the brain [60,62]. Two other methods may be operating to a lesser degree; the nanoparticles could pry open the tight junctions between cells to allow drugs to pass or the polysorbate-80 could inhibit the pump removal system [62]. 

It was mentioned above that the nanoparticles need to avoid sequestration by the body in order to be effective. This is normally achieved by creating a nanoparticle with a surface coating that can also act to stabilize the system. Poloxamine and poloxamer are often used as coatings because they avoid capture by the reticuloendothelial system. Work by Vila *et al*. compared three coatings that were intended to help overcome mucosal barriers as well as prevent uptake by the body [65]. Their three systems were poly(ethylene glycol)-coated poly(lactic acid) nanoparticles, chitosan-coated poly(lactic acid-glycolic acid) nanoparticles, and chitosan nanoparticles. A surface coating consisting of polyethylene glycol is hypothesized to hinder protein and enzyme adsorption onto the particle until it reaches its destination [65]. Chitosan was chosen based on qualities that have been mentioned before, such as its biodegradability and its innate ability to enhance transport of drugs across mucosal barriers [65]. Finally, the chitosan nanoparticle was conceived of as a solution to the use of organic solvents and energy-intensive methods of nanoparticle formation [65]. They observed that the nanoparticle size was easily tunable from 100 to 1000 nm, the maximum encapsulation efficiency was 33%, and saw improved protein stability and reduced aggregation for the poly(ethylene glycol)-coated nanoparticles [65]. The chitosan coating was also found to prevent aggregation and demonstrated encapsulation efficiency, however, it was less effective at transporting the drug to its desired location. The chitosan nanoparticles exhibited the same improved encapsulation efficiency with values as high as 50%, sizes between 300 and 400 nm, good transport through mucosal layers, and offer the additional benefit of immunostimulatory properties [65]. Thus, no formulation was objectively superior to the other and the choice of nanoparticle should be based on the individual system.

More research on this was done by Leroux *et al*., who made poly(DL-lactic acid) nanoparticles that could evade the mononuclear phagocyte system and deliver their drug over a 30 day period [66]. This was achieved by coating the surface with poly(ethylene glycol) which, in addition to hindering adsorption onto the surface, also extends in a brush formation to sterically stabilize the colloidal particles [66]. Work has also been done to incorporate a drug releasing layer into biodegradable films for coatings. Song *et al*. created a bilayer film in which one side contained the drug and the other was neat poly(lactic-co-glycolic acid) [61]. They found that the drug eluted only from the uncovered side and that the polymer side acted as a sealant against drug loss which could last for as long as two weeks [61]. This bilayer structure allows for more specialized distribution of the incorporated drug and could reduce the amount of material required by delivering it directly to the site of interest.

Chitosan has been incorporated into other systems as well that look to take advantage of its ability to entrap macromolecules through mechanisms such as ionic crosslinking, desolvation, and ionic complexation. One of the concerns in gene delivery is the toxicity of the polymers involved and so research has been done on creating DNA-chitosan hybrid nanospheres that function as transfection carriers [67]. Janes *et al*. used chitosan that had been gelled with polyanions to create chitosan nanoparticles with a coating of diblock copolymer poly(ethylene oxide) and poly(propylene oxide) [67]. They have found that chitosan-DNA complexes are based on strong charge interactions that prevent the complex from dissociating until it has entered the cell, but these have lower transfection efficacies than desirable. To solve this problem, they developed instead chitosan nanospheres containing DNA that were able to successfully transport it to the cell [67]. However, the gene expression levels are still too low and so they are currently only recommended for applications such as vaccinations [67].

Proteins also present a unique challenge to typical processing methods because they generally cannot survive being dissolved in an organic solvent and therefore cannot be loaded into nanoparticles by the previously described methods [68]. They also tend to be unstable in contact with gastrointestinal fluids and cannot be transported across mucosal barriers [65]. One solution has been presented by Blanco and Alonso who created poly(lactide-co-glycolide) nanospheres with improved uptake via M cells overtop Peyer’s patches [68]. They used a water-in-oil-in-water emulsion solvent evaporation technique with a minimal amount of organic solvent and increased sonication to produce spheres between 300 and 500 nm diameter [68]. Though their drug loading was highly variable, they were able to successfully transport hydrophilic proteins via nanospheres for the first time [68]. They then concluded that the efficiency was directly related to the polymer molecular weight, hydrophilicity, and the presence of poloxamer as a stabilizer, and the release rate of the drug was related to polymer hydrophilicity [68]. Janes *et al*. also found that they could use chitosan nanoparticles for the encapsulation of hydrophobic proteins by using a polar solvent to precipitate the peptide as nanocrystals within the nanoparticles [67]. These nanoparticles were observed to have protein loadings as high as 50%, which was attributed to a combination of physical entrapment during gelation, hydrophobic interactions, and hydrogen bonding [67]. Nihant *et al*. point out that the success of these encapsulated proteins depends on the kinetics of the main coacervation process which are determined by the volume of the drug-containing phase, the polymer addition rate, the stirring speed, and the encapsulated drug itself [69]. They found that particles tended to deform when the relative amount of the aqueous drug-containing phase increased and attributed this to the necessity of forming a thinner polymer coating in order to encapsulate each droplet [69]. Nihant *et al*. also found that the polymer precipitation rate was important with slower phase separation resulting in a more uniform size distribution and smooth particle surfaces as it allowed the system to get closer to equilibrium [69]. By decreasing the stirring speed, they identified a critical speed below which the droplets grow too large to be coated and become unstable [69]. Each of these is therefore important to keep in mind when optimizing a coacervation system.

## 4. Conclusions

From these four fascinating categories of materials, we can conclude that length scale is a crucial concern when processing nanomaterials for life science applications. Organization and structure on the microscale reflects the structure of the nanoscale as well and changes on one level will be sure to impact the other. As microscopy and processing techniques continue to improve and expand our access to the nano-world, familiar and novel materials must be tuned to reflect that. Nacre is an ideal biomimetic material for enhancing composite strength through clever orientation of components rather than stronger building blocks. By looking at features on the nanoscale such as asperities and organic-inorganic layer interactions, we can elucidate stress mechanisms worth incorporating into engineered devices. Hydroxyapatite fibers are an example of how acknowledgement of the nanoscale can improve preexisting materials as previously engineered materials were detailed to the microscale, often without nanoscale features. However, in nature, collagen fibers are studded with nanocrystals of hydroxyapatite and the addition of nano-details to synthetic hydroxyapatite and polymer composites dramatically increased their ability to integrate with the body. Nanosized particles allow for greater freedom to create composites because their inclusion does not have to impact the structure. Bioglass offers very desirable bioactivity, but part of its appeal is certainly that it can be incorporated into preexisting materials such as foam scaffolds without negatively affecting the desired structure. The same is true of the catalyst nanoparticles described above which allow polymers to self-heal. Because of their small size, relatively high concentrations can be incorporated without interfering with the structure. Lastly, the creation of nanoparticles allows novel transport of engineered drugs through the blood-brain barrier when before microparticles were too large to even reach the epithelial cells. Therefore, these four examples and more demonstrate the importance of the nanoscale with regard to materials engineering. All of these materials demonstrate unique structuring on every length scale from the macro- to the nano-level, and as a result of this hierarchy they have improved material properties and homogeneity across large areas. Each length scale is individually equipped to handle stresses and deformation behavior in one layer is tied to the next layer in order to prevent damage. When engineering our composites it is essential that we do the same and optimize material interactions even at the nano-level.
